# Improved protocol for metabolite extraction and identification of respiratory quinones in extremophilic Archaea grown on mineral materials

**DOI:** 10.3389/fmicb.2024.1473270

**Published:** 2025-01-08

**Authors:** Sebastian V. Gfellner, Cyril Colas, Guillaume Gabant, Janina Groninga, Martine Cadene, Tetyana Milojevic

**Affiliations:** ^1^UPR4301 Centre de Biophysique Moléculaire (CBM), Orléans, France; ^2^Université d'Orléans, Orléans, France; ^3^UMR7311 Institut de Chimie Organique et Analytique (ICOA), Orléans, France; ^4^Center for Marine Environmental Sciences, University of Bremen, Bremen, Germany

**Keywords:** metabolomics, chemolithotrophs, thiophene-bearing quinones, quorum sensing, organic extraction from minerals

## Abstract

We investigated the metabolome of the iron- and sulfur-oxidizing, extremely thermoacidophilic archaeon *Metallosphaera sedula* grown on mineral pyrite (FeS_2_). The extraction of organic materials from these microorganisms is a major challenge because of the tight contact and interaction between cells and mineral materials. Therefore, we applied an improved protocol to break the microbial cells and separate their organic constituents from the mineral surface, to extract lipophilic compounds through liquid–liquid extraction, and performed metabolomics analyses using MALDI-TOF MS and UHPLC-UHR-Q/TOF. Using this approach, we identified several molecules involved in central carbon metabolism and in the modified Entner-Doudoroff pathway found in Archaea, sulfur metabolism-related compounds, and molecules involved in the adaptation of *M. sedula* to extreme environments, such as metal tolerance and acid resistance. Furthermore, we identified molecules involved in microbial interactions, i.e., cell surface interactions through biofilm formation and cell–cell interactions through quorum sensing, which relies on messenger molecules for microbial communication. Moreover, we successfully extracted and identified different saturated thiophene-bearing quinones using software for advanced compound identification (MetaboScape). These quinones are respiratory chain electron carriers in *M. sedula*, with biomarker potential for life detection in extreme environmental conditions.

## 1 Introduction

Analogous to the first microorganisms that inhabited the early Earth, chemolithoautotrophic microorganisms use ancient metabolic pathways to harvest energy either through mineral redox alterations, or directly from inorganic compounds, such as nitrogen, iron, or sulfur (Vargas et al., 1998; Wächtershäuser, 1988, 1990; Weiss et al., 2016; Camprubi et al., 2017; Morrison and Mojzsis, 2021). Archaea from the order Sulfolobales (e.g., *Sulfolobus* spp., *Acidianus* spp. and *Metallosphaera* spp.) are capable of oxidizing Fe and S while thriving under extreme conditions at a pH of 2–3 and temperatures of 65–80°C. These microorganisms can use heterotrophic, chemolithoautotrophic, and mixotrophic ways of generating energy utilizing various substrates, such as complex organic molecules, CO_2_ fixation, and oxidation of various metal sulfides (Huber et al., 1989; Clark et al., 1993; Peeples and Kelly, 1995; Schönheit and Schäfer, 1995; Amend and Shock, 2001; Auernik et al., 2008; Auernik and Kelly, 2008, 2010a, 2010b; Maezato et al., 2012; Mukherjee et al., 2012; Kölbl et al., 2017; Wheaton et al., 2019; Blazevic et al., 2019; Milojevic et al., 2021).

The archaeon *Metallosphaera sedula* is known for its potential to mobilize metal sulfides and oxides and a broad range of mineral biotransforming capabilities, which span from Fe and S minerals, such as pyrite FeS_2_ (Clark et al., 1993; Schönheit and Schäfer, 1995; Amend and Shock, 2001) and chalcopyrite CuFeS_2_ (Maezato et al., 2012), to calcium tungstate minerals such as scheelite (Blazevic et al., 2019), uranium (Mukherjee et al., 2012), molybdenum, and vanadium (Wheaton et al., 2019). *M. sedula* was isolated from a sulfataric field in Italy by Huber et al. (1989), and its fully sequenced genome has been intensively studied, with a focus on its bioleaching capabilities (Auernik et al., 2008; Auernik and Kelly, 2008). Moreover, Kölbl et al. (2017) focused on the utilization of extraterrestrial material by *M. sedula* grown on Martian regolith simulants, paving the way for the cultivation of *M. sedula* on the genuine Martian meteorite NWA 7034 by Milojevic et al. (2020).

**Figure 1:**
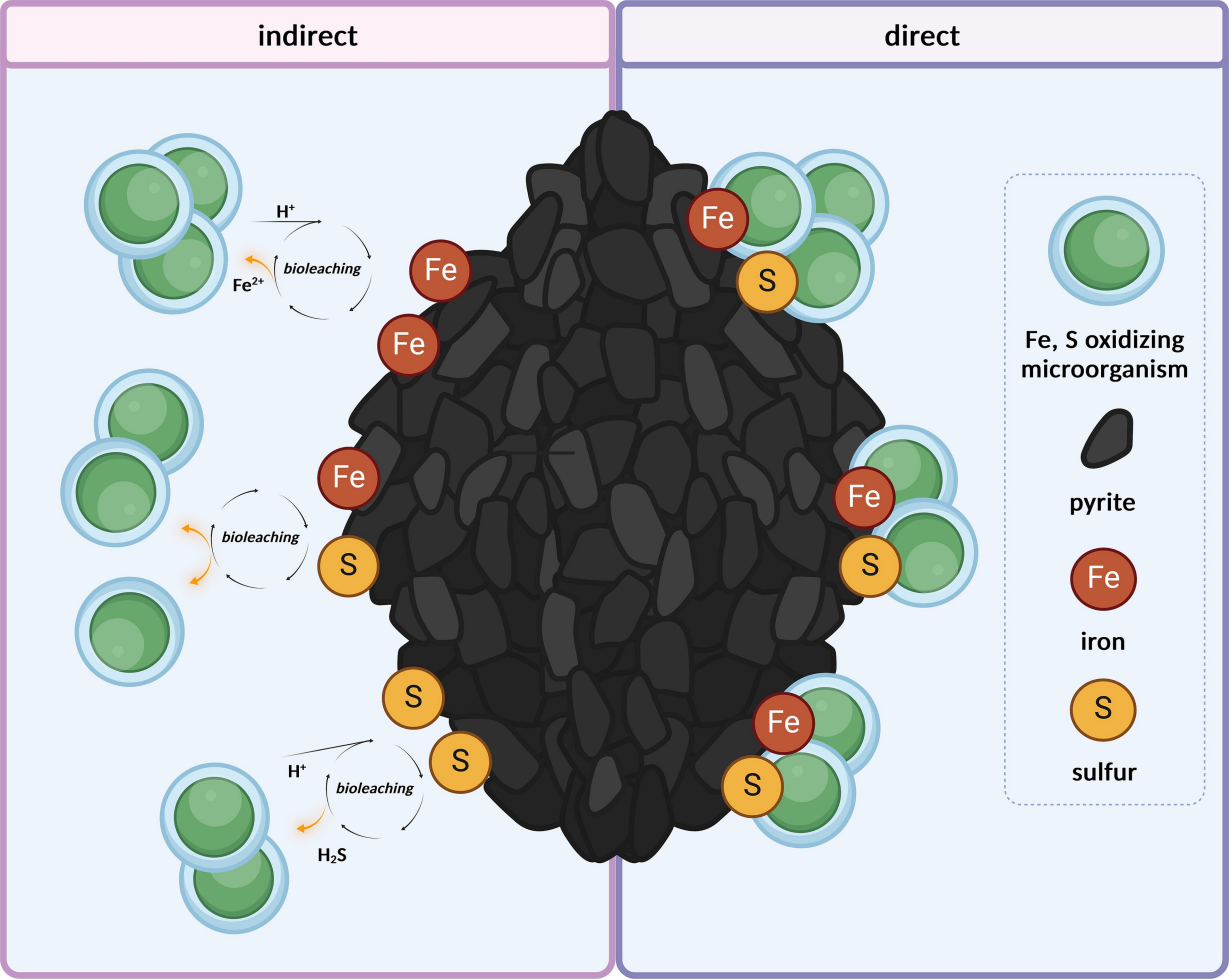
Microbial harvest of energy through the oxidation of Fe^2+^ and reduced inorganic sulfur compounds from mineral matrix, either indirectly from ions released in the medium through abiotic acidic leaching (under a pH of 2.0) or directly from the mineral surface. Created with BioRender.com.

Bioleaching involves the oxidation of metals and metalloids, accompanied by the release of metal compounds from the mineral matrix (Figure 1); for example, Fe(II) is oxidized to Fe(III). Remarkable is the cellular resistance of acidophilic microorganisms (e.g., *M. sedula*) to heavy metals such as As, Cu, Zn, Cd, and Ni, as reviewed by Dopson et al. (2003). The multimolecular machinery of iron-transforming Archaea is usually represented by clusters of redox-active enzymes associated with respiratory Fe/S oxidation. For instance, for Fe-oxidizing members of the archaeal order Sulfolobales, the existence of the ferrous iron oxidation (fox) gene cluster has been reported (Counts et al., 2022), the products of which include the primary electron acceptor from metal ions and terminal oxidase complex. The surface attachment of mineral-transforming microorganisms and biofilm formation on mineral surfaces are crucial strategies that enhance microbial bioleaching performance and facilitate mineral solubilization, with increasing interest in industrial applications (Rohwerder et al., 2003; Olson et al., 2003). Biofilm formation in Sulfolobales involving attachment, maturation, and dispersal has been described, whereas the bioalteration of mineral materials with extracellular matrices composed of carbohydrates has been observed (Koerdt et al., 2010, 2012; Lewis et al., 2023). The adsorption of microorganisms onto mineral surfaces occurs locally in low-pH microenvironments containing extracellular polymers (Xia et al., 2021). The role of extracellular polymeric substances, mainly neutral sugars and lipids, in attachment to mineral surfaces such as pyrite has been further investigated by Kinzler et al. (2003), focusing on the bacteria *Acidithibacillus ferrooxidans*, which mediates attachment to the sulfide surface and concentration of Fe due to complexation promoting sulfide oxidation. This was also consistent with the studies by Bromfield et al. (2011), who investigated the mineral adsorption of the extreme thermoacidophilic archaeon *M. hakonensis* onto mineral sulfides, underlining the importance of surface charge rather than hydrophobic interactions.

**Figure 2:**
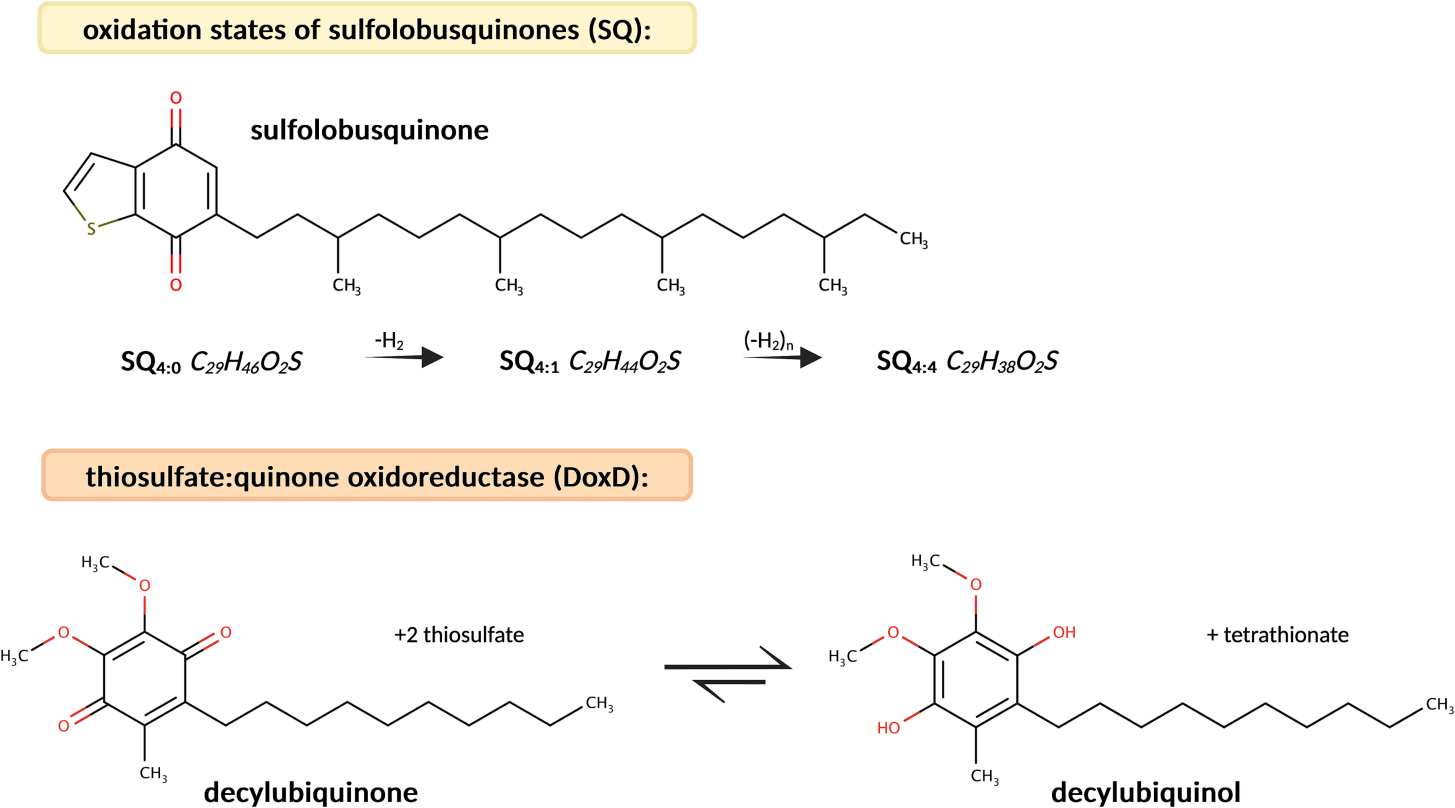
Molecular structure of sulfolobusquinone (SQ) and molecular formulas of its different oxidation states (SQ_4:0_, SQ_4:1_, and SQ_4:4_). The reaction pathway of decylubiquinone to decylubiquinol though catalysis by thiosulfate:quinone oxidoreductase (DoxD) is described in the KEGG database.

The detailed biochemical processes involved in the metabolism of *M. sedula* underlying the oxidation of iron and sulfur compounds in the order of Sulfolobales have been described by transcriptome analyses of *M. sedula* grown on various mineral substrates, enabling the comprehensive identification of its electron transport chains (Auernik et al., 2008; Auernik and Kelly, 2008, 2010a, 2010b). This includes the distinct role of isoprenoid quinones, which are part of the membranes of all living organisms (Hiraishi, 1999). They are composed of a hydrophilic head group and an apolar isoprenoid side chain. Therefore, they exhibit amphiphilic properties, which allows them to insert into lipid bilayers. They mainly function as electron and proton carriers in photosynthetic and respiratory electron transport chains, with additional roles as antioxidants (Hiraishi, 1999; Nowicka and Kruk, 2010). Quinone oxidoreductases deliver electrons to terminal oxidase complexes that maintain intracellular pH while generating a proton motive force (thiosulfate:quinone oxidoreductase, e.g., DoxD) through reduced caldariella- or sulfolobus-type quinones (Figure 2) (Auernik and Kelly, 2008). Furthermore, the role of signaling molecules involved in quorum sensing has been intensively investigated, as this cell–cell communication strategy enables cross-species microorganisms to synchronize their gene expression and growth (Ng et al., 2011; Hiblot et al., 2012; Kaur et al., 2018). To date, the best-characterized signaling molecules in Archaea are N-acyl homoserine lactones (AHLs), which potentially promote biofilm formation and, therefore, play an important role in microbe-mineral interactions (Charlesworth et al., 2020; Prescott and Decho, 2020). Additionally, quorum-sensing molecules have been proposed as biocatalysts to increase microbial turnover rates during biomining operations (Ruiz et al., 2008; Bellenberg et al., 2014).

Although the efficient separation of microbial organic molecules from mineral materials is challenging (Direito et al., 2012; Swenson et al., 2015; Swenson and Northen, 2019; Bell et al., 2022), the metabolomics of microbial-mineral interactions is a promising and powerful tool for microbial screening of environmental samples for a number of biotechnological and potential astrobiological applications (Giebel et al., 2010; Das and Dash, 2014; Röling et al., 2015; Abrahamsson and Kanik, 2022; Sharma et al., 2022). However, separation of biologically active molecules from mineral matrices remains problematic because of the strong adsorption of organic substances by iron-rich minerals (Direito et al., 2012; Röling et al., 2015; Swenson et al., 2015; Swenson and Northen, 2019; Abrahamsson and Kanik, 2022; Bell et al., 2022). In this study, we report the successful extraction of metabolites from the chemolithotrophic organism *M. sedula* grown on a mineral source, by adapting a modified lipid extraction protocol and implementing mass spectrometry-based metabolomic analysis. This mass spectrometry-based technique can be further applied to detect thiophene-bearing quinones in environmental and laboratory samples, to resolve metabolic pathway-specific molecules, and to provide insight into the metabolome used in microbe-mineral interactions in *M. sedula*.

## 2 Materials and methods

### 2.1 Microbial cultivation

*Metallosphaera sedula* DSM 5348 was cultivated aerobically in DSMZ 88 medium in the presence of pyrite, as described previously (Kölbl et al., 2017; Blazevic et al., 2019; Milojevic et al., 2021), over a period of 140 h in 1 L glassblower modified Schott-bottle bioreactors (Duran DWK Life Sciences GmbH, Wertheim/Main, Germany), unless otherwise noted. The stock culture was stored at −80°C in a mixture of 50% glycerol and DSMZ 88 medium (50, 50, v:v). The DSMZ 88 medium is composed of 9.84 mM (NH_4_)_2_SO_4_, 2.06 mM KH_2_PO_4_, 1.01 mM MgSO_4_ × 7H_2_O, 0.48 mM CaCl_2_ × 2H_2_O, and 0.07 mM FeCl_3_ × 6H_2_O. This was also used as cell resuspension medium. Further, Allen trace element solution was added consisting of 0.91 mM MnCl_2_ × 4H_2_O, 1.18 mM Na_2_B_4_O_7_ × 10H_2_O, 0.08 mM ZnSO_4_ × 7H_2_O, 0.03 mM CuCl_2_ × 2H_2_O, 0.01 mM Na_2_MoO_4_ × 2H_2_O, 0.02 mM VOSO_4_ × 2H_2_O, and 3.56 μM CoSO_4_ × 7H_2_O. Tryptone (0.1%) was added to the DSMZ 88 medium, as previously described (Kölbl et al., 2017). The pH was adjusted to 2.0, with 5 M H_2_SO_4_. The pyrite was manually ground using a hand grinder to particles with diameters of 63–100 μm, controlled by 63 μm and 100 μm mesh sieves with a 75:25% distribution of 63 to 100 μm, and baked overnight at 180°C. Pyrite (10 g/L) was added to 800 mL of culture. A 1 L bioreactor was then assembled as described previously (Kölbl et al., 2017; Blazevic et al., 2019; Milojevic et al., 2021) and constantly heated to 73°C with steady stirring. A flow of CO_2_ at a total rate of 0.9 L/min (normalized to 1 atm and 0°C) was ensured for the interconnected triplicate bioreactor setup, resulting in a flow rate of 0.3 L/min for each bioreactor. Three biological replicates (A, B, and C) were incubated and harvested before reaching the stationary phase. For inoculation, a frozen (−80°C) glycerol stock of *M. sedula*, previously grown and acclimated to pyrite, was used. To monitor microbial growth, the cultures were sampled continuously during the growth phase and the cells were counted under a microscope (Olympus BX51 equipped with a Pixelink M20C-CYL camera) using a Neubauer Chamber (Carl Roth GmbH & Co. KG, Karlsruhe, Germany) and harvested upon reaching stationary phase. Harvesting was performed by centrifugation in sterile 50 mL Falcon tubes at 3220 × *g* for 40 min. The cell pellets and supernatants were collected separately, snap-frozen in liquid nitrogen, and stored at −20°C until further extraction.

### 2.2 Lyophilization and hydrolysis

The stored cell/pyrite pellets were thawed and resuspended in cell resuspension medium and transferred into a 50 mL glass vial. The samples were then refrozen and lyophilized overnight. To increase the detection capabilities and promote the separation of cells and minerals, the samples were hydrolyzed before extraction using 1 M HCl in a mixture of methanol (1:1, v/v), and vortexed and ultrasonicated for 10 min. The mixture was then heated to 70°C for 3 h and dried under a stream of N_2_ at 60°C. This will be referred to as hydrolyzed cells/pyrite.

### 2.3 Biomass extraction: cell breakage and liquid**–**liquid extraction

To increase the yield of metabolites, a protocol originally developed by Bligh and Dyer (1959) for total lipid extraction and modified by Evans et al. (2022) was used to separate organic molecules from the mineral phases. This protocol was applied to dried hydrolyzed cells/pyrite. To burst the microbial cells, separate the cell debris from the minerals and release metabolites into the solution, a volume of 5 mL B&DI solution consisting of methanol, dichloromethane, and 0.1 M potassium phosphate buffer, pH 8.0 (2:1:0.8, v/v/v) was added to 1 g pyrite-equivalent of hydrolyzed cells/pyrite samples. The samples were vortexed, sonicated for 10 min and centrifuged for 10 min at 3220 × *g* in a 50 mL glass vial. The supernatant was decanted into a fresh glass vial and evaporated under a stream of N_2_ at 60°C. This step was repeated once with B&DI and twice with B&DII consisting of methanol, dichloromethane, and 0.1 M trichloroacetic acid solution (2:1:0.8, v/v/v). This will be subsequently referred to as B&D extract. To enrich the lipophilic compounds, liquid–liquid extraction was conducted on the B&D extract in 50 mL glass vials. For this, 5 mL ultrapure water and 10 mL dichloromethane were added, followed by vortexing and centrifugation for 5 min at 3220 × *g*. The organic phase at the bottom of the vial was extracted into a fresh glass vial, and the 50 mL glass vials with the aqueous phase were set aside. This liquid–liquid extraction step was repeated four times and the organic fractions were pooled and evaporated under a stream of N_2_ at 60°C. The organic fraction of the extract was then transferred using 4 mL dichloromethane into a fresh vial, evaporated under a stream of N_2_ at 60°C and resolubilized with 200 μL of methanol and dichloromethane (9:1, v/v). To be able to separately analyze hydrophilic compounds, the aqueous phase in the 50 mL glass vials was further processed. To exclude lipophilic compounds from the aqueous phase, 10 mL of dichloromethane were added, followed by sonication for 10 min and centrifugation for 10 min at 3220 × *g*. Then, the aqueous fraction was collected, transferred with 4 mL of ultrapure water into a fresh vial, evaporated under a stream of N_2_ at 60°C and resolubilized with 200 μL of ultrapure water and methanol (9:1, v/v).

### 2.4 MALDI-TOF mass spectrometry

The exometabolites in *M. sedula* were identified by comparative Matrix Assisted Laser Desorption Ionization – Time of Flight mass spectrometry (MALDI-TOF MS) analysis of a 4.5 L culture of *M. sedula* grown on pyrite. Analysis was conducted of the cell pellet, the corresponding culture supernatant, and the organic extract. MALDI-TOF MS spectra were acquired on an ultrafleXtreme mass spectrometer (Bruker Daltonics GmbH, Bremen, Germany). Samples were mixed in a 1:1 ratio in a solution consisting either of 4-hydroxy-*α*-cyano-cinnamic acid (HCCA) or 2,5-dihydroxybenzoic acid (DHB) at 1 mg/mL in acetonitrile, ultrapure water, and trifluoroacetic acid (50:47.5:2.5, v/v/v) containing 1 mM NaCl. The matrix-sample solutions were spotted onto an AnchorChip target and air-dried. Spectra were acquired in reflectron positive ion mode (3,000 laser shots) in the 100–4,500 *m/z* range. Calibration of the instrument was performed externally using a neighboring spot with peaks of the matrix and pepmix calibration standard II (Bruker Daltonik GmbH, Bremen, Germany), with the addition of oxidized insulin B and adrenocorticotropic hormones (clip 1–39). MALDI-TOF MS spectra were processed using the FlexAnalysis v3.4 software (Bruker). After mass spectral comparison, statistical analysis revealed shared masses and masses present only in the cell pellet, the culture supernatant, and the organic extract.

### 2.5 UHPLC-UHR-Q/TOF mass spectrometry

Analyses were performed using an UltiMate 3000 RSLC system (Dionex, Germering, Germany) connected to a maXis ultra-high resolution quadrupole-TOF mass spectrometer (UHR-Q/TOF MS) (Bruker Daltonics, Bremen, Germany) equipped with an electrospray ion source. Metabolites were separated on an Acquity UPLC BEH C18 1.7 μm 2.1 × 100 mm column (Waters, Saint-Quentin-en-Yvelines, France). For the analysis of the organic phase of the extract, the column was heated at 60°C and the following solvents were used at a flow rate of 500 μL/min: H_2_O with 0.1% formic acid as solvent A and a mixture of methanol and isopropanol (50:50, v/v) with 0.1% formic acid as solvent B. Gradient elution was set to 0–2.5 min, 3% B; 2.5–4 min, 6% B; 4–13 min, 85% B; 13.5–19.1 min, 100% B; and 19.1–23 min, 3% B. A volume of 2 μL of the organic extract was injected. To analyze the aqueous phase of the extract, the column was heated at 40°C, and the following solvents were used at a flow rate of 500 μL/min: H_2_O with 0.1% formic acid as solvent A and acetonitrile with 0.08% formic acid as solvent B. Gradient elution was set to 0–10 min, 3% B; 10–13 min, 45% B; 13–15 min, 100% B; and 15–18 min, 3% B. A volume of 0.5 μL of the aqueous extract was injected. Mass spectra were acquired in positive ion mode at a frequency of 1 Hz in the 50–1,650 *m/z* range. The ESI source parameters were as follows: nebulizing gas, 2 bar; drying gas, 200°C at a flow rate of 9 L/min; capillary voltage, 4,500 V.

### 2.6 Data analysis and advanced data processing using MetaboScape®

Data processing was performed using DataAnalysis 4.4 software (Bruker Daltonics, Bremen, Germany). Lock mass calibration was performed at *m/z* 622.0296 [hexakis(2,2-difluoroethoxy)phosphazine; CAS #:186817–57-2], and the peaks (*m/z*) were identified based on mass accuracy, isotope patterns, and retention time. The metabolite analysis was based on the translation of the KEGG pathways of *M. sedula* into target molecules. The purified total lipid extract from Elling et al. (2016) was previously used to identify S-bearing quinones in Sulfolobales, and a database containing the theoretical *m/z* of different oxidation forms of sulfulobusquinones (SQ), caldariellaquinones (CQ), and benzodithiophenequinones (BDTQ) was kindly provided by Felix J. Elling (Leibniz-Laboratory for Radiometric Dating and Isotope Research, Christian-Albrecht University of Kiel, Germany). To verify the feasibility of the method for quinone detection, we applied the quinone detection protocol described by Elling et al. (2016), which was initially reproduced for *S. acidocaldarius* and subsequently adapted it to *M. sedula*. The peaks were then integrated, and the area under the curve (AUC) of the corresponding annotated molecules was compared in biological triplicates, with each biological replicate measured in technical triplicates alongside an additional blank. Further advanced data analysis was conducted using MetaboScape 2024b® (Bruker Daltonics, Bremen, Germany) with its embedded T-ReX® feature finder algorithm, which encompasses retention-time alignment, mass calibration, and peak picking. For feature finding, we set an intensity threshold of 10,000 counts, a minimum peak length of six spectra, and enabled the recursive feature-finding tool to achieve high and reliable coverage. For ion deconvolution, [M + H]^+^ was set as the primary ion and [M + NH_4_]^+^ and [M + Na]^+^ were set as potential seed ions. Data filtering ensured that only the features present in at least two samples were recognized and extracted. To automatically annotate our target compounds, we defined and uploaded a target list of sulfur-bearing quinones and performed annotation with a mass tolerance of 2 ppm and a maximum mSigma value (isotopic pattern fit) of 40.

## 3 Results and discussion

### 3.1 Microbial cultivation of *Metallosphaera sedula* on pyrite

The *M. sedula* cultures were inoculated in a suspension of pyrite in pH 2.0 growth medium supplemented with air and CO_2_ in triplicate bioreactors (A, B, C) as described in the methods section. *M. sedula* cultures were harvested before reaching stationary phase (Supplementary Figure S1).

The cell densities from inoculation to harvest were equivalent in all four bioreactors (Table 1).

**Table 1:** Cell densities [cells/mL] of *n* = 3 biological replicates (A, B, and C) of *Metallosphaera sedula* grown on pyrite (10 g/L) at inoculation time point (*t* = 0 h) and harvest time points (*t* = 140 h; *t* = 312 h).

Replicate	Cell density [cells/mL]		
	*t* = 0 h	*t* = 140 h	*t* = 312 h
Initial culture	7.89 × 10^6^ ± 3.88 × 10^6^		3.37 × 10^7^ ± 2.43 × 10^6^
A	4.71 × 10^6^ ± 2.55 × 10^5^	1.14 × 10^7^ ± 2.78 × 10^5^	
B	5.33 × 10^6^ ± 3.80 × 10^3^	1.08 × 10^7^ ± 2.56 × 10^4^	
C	5.15 × 10^6^ ± 2.91 × 10^4^	9.78 × 10^6^ ± 1.07 × 10^5^	

### 3.2 Metabolomic profiling of *Metallosphaera sedula* grown on pyrite

To separate the organic molecules from the mineral pyrite material, the Bligh and Dyer protocol (Bligh and Dyer, 1959) modified by Evans et al. (2022) was applied. Evans et al. concluded that the yield of archaeal lipid extraction was higher with trichloroacetic acid solution than with sodium phosphate buffer. Since potassium phosphate buffer has the potential to chelate soluble metal ions better than sodium phosphate buffer, we further improved the modified Bligh and Dyer protocol by using a potassium phosphate buffer solution (KH_2_PO_4_). In order to cover a wider range of metabolites, we also decided to alternate acidic and alkaline extraction steps. To enrich the lipophilic compounds, a liquid–liquid extraction step was added to the protocol and the analysis of hydrophilic compounds was made possible by a clean-up step of the aqueous fraction. This additional step was particularly necessary for amino acids.

**Figure 3:**
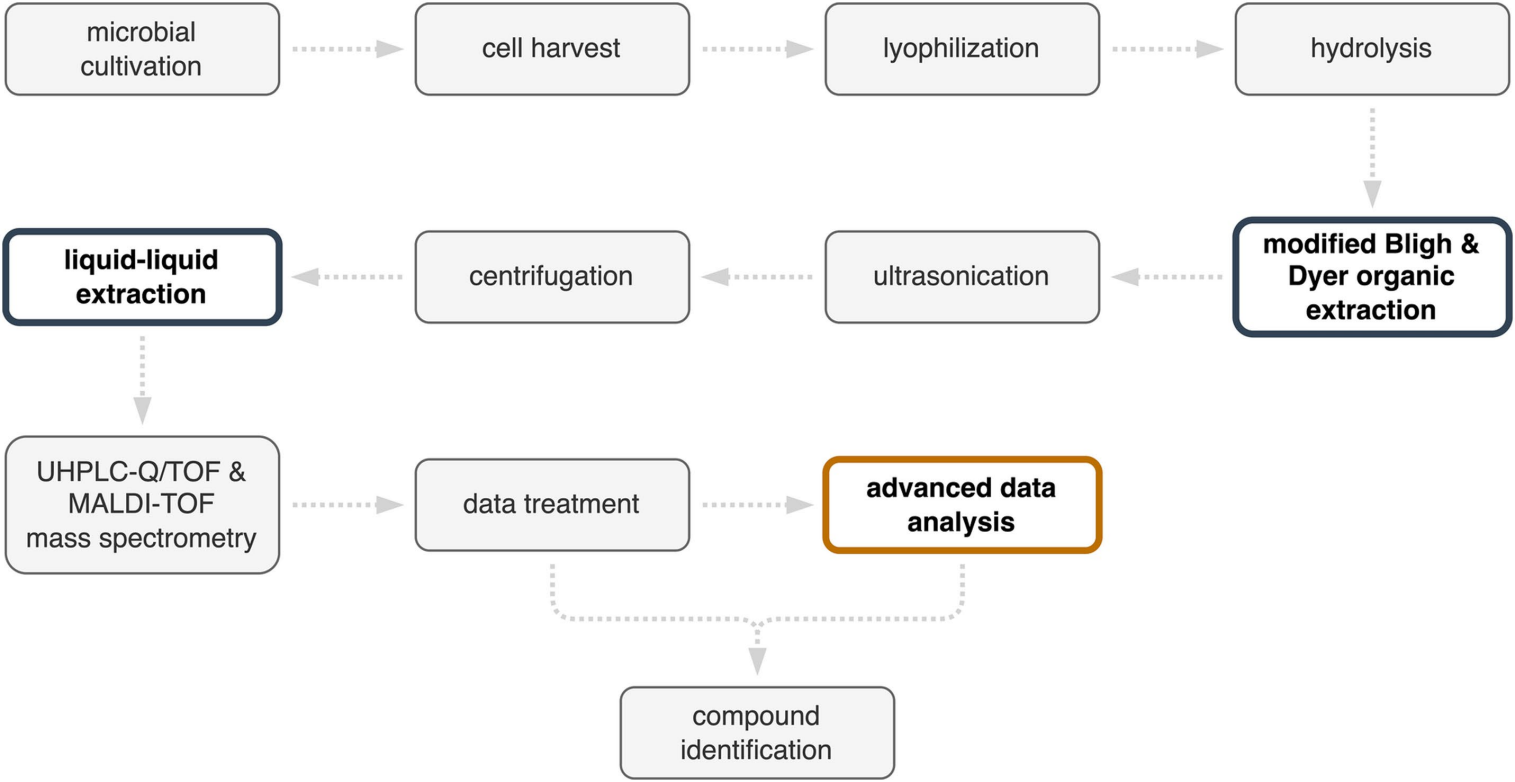
Protocol for the identification of *Metallosphaera sedula* metabolites from cells cultivated in a pyrite suspension medium. Improvements in the protocol are highlighted in color.

The protocol for the identification of metabolites, from microbial cultivation to compound identification, is presented in the flowchart in Figure 3. The efficiency of compound identification was increased by combining a reference database with MetaboScape®, an advanced data analysis software. This protocol also has the potential for extracting metabolites from other members of the order of Sulfolobales in the presence of mineral substrates. Indeed, the Bligh and Dyer protocol was developed to separate organic material from mineral materials, and has been used for the extraction of organics from deep-sea sediments to microbial mat systems. In the present study, the quantities of solvents used for Bligh and Dyer extraction were selected based on 1 g of pyrite equivalent of sample material. However, the ability of the protocol to extract compounds may be limited by the solubility of different target molecules. This could be circumvented by adapting the solvents chosen for liquid–liquid extraction.

#### 3.2.1 Distinguishing between metabolites and exometabolites via profiling using MALDI-TOF mass spectrometry

Comparative analysis of the culture supernatant, cell pellet, and organic extract by MALDI-TOF MS can help differentiate between metabolites in the cell and those released into the culture medium, i.e., between metabolites and exometabolites. MALDI-TOF MS analysis is relatively fast to implement and was used as a preliminary step to evaluate the presence of compounds in these fractions. To obtain initial metabolomics data on mineral-adapted *M. sedula*, the microorganism was grown in a 4.5 L bioreactor over a timespan of 312 h. The culture was harvested after reaching the stationary phase under the previously described cultivation and extraction conditions. Comparative analysis using MALDI-TOF MS of culture supernatant, cell pellet, and organic extract revealed 116 measured *m/z* across all biological samples (Supplementary Table S1). 20 compounds were found only in the culture supernatant, 16 in the cell pellet analysis, 19 in the culture supernatant and cell pellet analysis, and 61 were found only in the organic extract. A metabolite screening allowed for the annotation of 13 compounds (Table 2). These annotations were confirmed by UHPLC-UHR-Q/TOF MS analysis. However, the compound lists obtained by MALDI-TOF MS in this initial experiment represent a restricted dataset due to limited sensitivity and dynamic range, which are attributed to competition with the HCCA matrix and the absence of prior HPLC separation. To explore the sample more in depth and gain a more comprehensive and coherent view of the *M. sedula* metabolome, an LC-ESI-based analysis was conducted using the same extraction protocol.

**Table 2:** Comparative analysis of intra- and extracellular metabolites by MALDI-TOF MS.

Biological sample	Measured compounds	Annotated compounds	Compound annotation
Culture supernatant	20	1	Leucylvaline
Cell pellet	16	0	
Culture supernatant and cell pellet	19	0	
Organic extract	61	12	Gln, Lys, Trp, Pyl, ribose, 2-deoxy-D-ribose, 2-deoxy-D-glucose, hexose, D-glucosamine/galactosamine, phosphoenolpyruvate, 2-dehydro-3-deoxy-6-phospho-D-gluconate, 2-keto-3-deoxygluconate, decylubiquinol

#### 3.2.2 UHPLC-UHR-Q/TOF mass spectrometry

**Figure 4:**
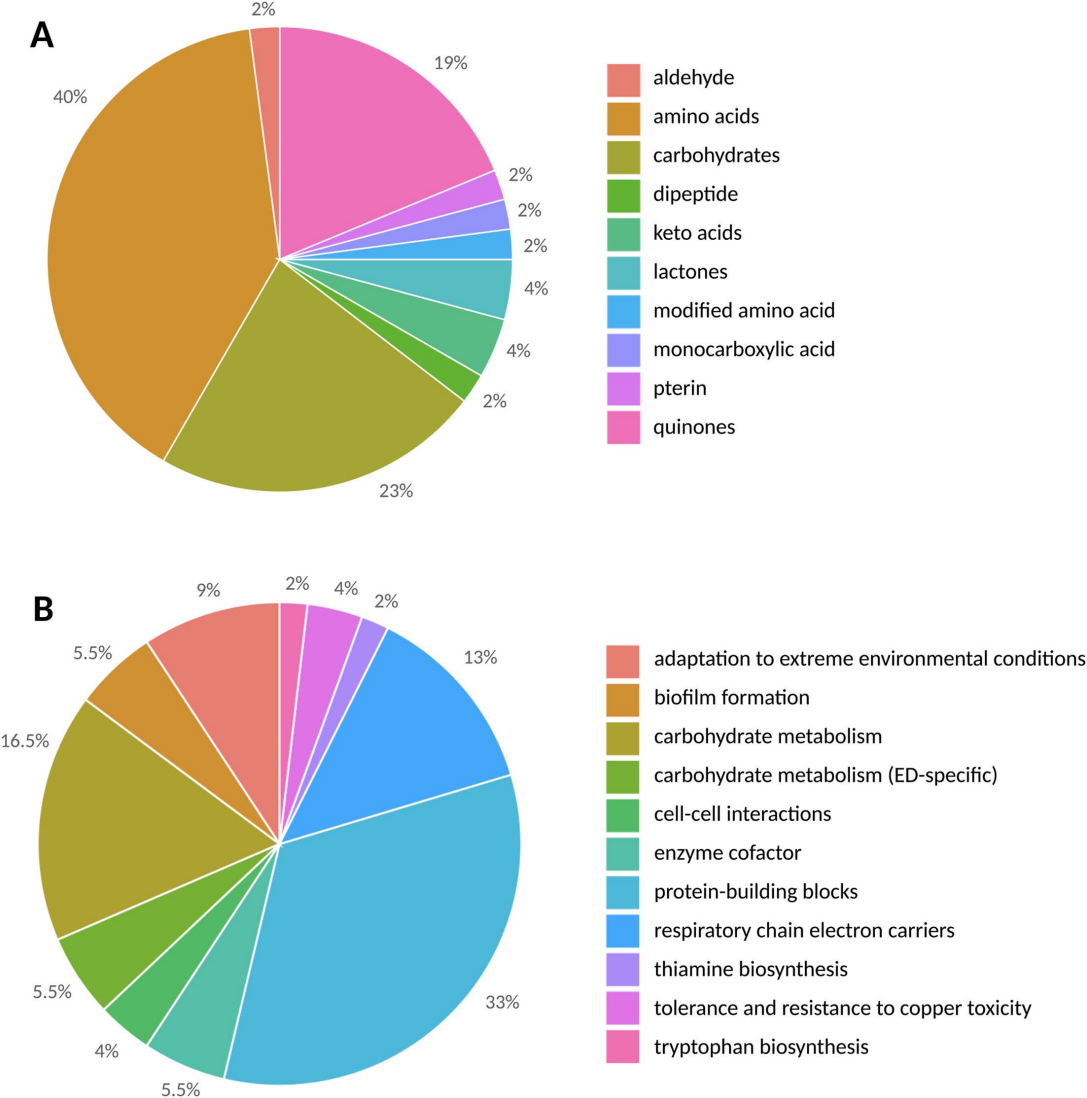
The structural **(A)** and functional **(B)** categories of metabolites in *Metallosphaera sedula* triplicates are represented by the number of detected metabolites.

Data annotation and treatment for compound identification for metabolomic profiling was conducted using the DataAnalysis software. For the identification of thiophene-bearing quinone, the advanced data analysis software MetaboScape® was used for molecular assignment. Metabolite analysis by UHPLC-UHR-Q/TOF MS of organic and aqueous fractions of the extracts revealed 48 metabolites (Supplementary Table S2). Based on the results obtained, the metabolites identified through ESI-based analysis were grouped into structural and functional categories (Figure 4).

Structurally (Figure 4A), the highest number of metabolites was found in the amino acid group, with a total number of 19, followed by 11 carbohydrates, 2 keto acids, 2 lactones, 1 monocarboxylic acid, 1 pterin, 1 dipeptide, 1 aldehyde, 1 modified amino acid, and 9 quinones. However, as the medium contains 0.1% tryptone containing 18 amino acids (Ala, Arg, Asp., Cys, Glu, Gln, His, Leu/Ile, Lys, Met, Phe, Pro, Ser, Thr, Trp, Tyr, and Val), of the identified amino acids in this study, only Asn and Pyl of can be unambiguously ascribed to the contribution of *M. sedula*.

The metabolites were also grouped into functional categories (Figure 4B; Table 3). Their biological functions span from energetic metabolism, anabolism/catabolism, to biofilm formation, cell–cell interactions, and metal complexation.

**Table 3:** Grouping of metabolites identified in the present study into functional categories.

Metabolite	Compound class	Functional category
Arg, Asn, Asp., Cys, Glu, Gln, His, Ile/Leu, Lys, Met, Phe, Pro, Ser, Thr, Trp, Tyr, Val, Pyl	Amino acid	Protein-building blocks
His, Met	Amino acid	Tolerance and resistance to copper toxicity
leucylvaline, 8-amino-7-oxononanoic acid, N-acetyl-D-glucosamine/galactosamine^*^, N-acetylmuramic acid, S-adenosylmethionine	Dipeptide, amino acid, carbohydrate, modified amino acid	Adaptation to extreme environmental conditions
D-glyceraldehyde-3-phosphate, 2-dehydro-3-deoxy-6-phospho-D-gluconate, phosphoenolpyruvate, 1,3-bisphosphoglycerate, D-glucosamine/galactosamine, D-glucuronic/galacturonic acid, ribose, 2-deoxy-D-ribose, 2-deoxy-D-glucose	Aldehyde, monocarboxylic acid, carbohydrate	Carbohydrate metabolism
D-gluconate/galactonate, 2-keto-3-deoxygluconate, 2-keto-3-deoxy-6-phosphogluconate	Keto acid	Carbohydrate metabolism (ed-specific)
D-glyceraldehyde-3-phosphate	Aldehyde	Tryptophan and thiamine biosynthesis
N-(3-oxohexanoyl)-L-homoserine lactone, lactone	Lactone	Cell–cell interactions
hexose, N-acetyl-D-glucosamine/galactosamine^*^, N-acetylmuramic acid	Carbohydrate	Biofilm formation
MoCo II, decylubiquinone, decylubiquinol	Pterin, quinone	Enzyme cofactor
CQ_4:1_, CQ_5:1_, SQ_4:0_, SQ_4:1_, SQ_5:0_, SQ_5:1_, BDTQ_5:0_	Quinone	Respiratory chain electron carriers

*Identified as N-acetyl-hexosamine.

The modified lipid extraction protocol applied in our study allowed us to identify a wide variety of metabolites and proved to be suitable for separating organics from the mineral material. The dataset also suggested the biological processes underlying these metabolites (Table 3).

Among the metabolites involved in carbon metabolism, the associated metabolites D-glyceraldehyde-3-phosphate, 2-dehydro-3-deoxy-6-phospho-D-gluconate, phosphoenolpyruvate, D-glucosamine/galactosamine, D-glucuronic/galacturonic acid, ribose, 2-deoxy-D-ribose, and 2-deoxy-D-glucose are generally involved in core carbon metabolism and carbohydrate degradation (Fisher, 2001; Bräsen et al., 2014). In contrast, 1,3-bisphosphoglycerate is involved in gluconeogenesis (Siebers and Schönheit, 2005). Since no glucose was added during cultivation, the only identifiable source for glucose-related metabolism might be remnants of glycerol. To store *M. sedula* cultures, we used a mixture of 50% glycerol and DSMZ 88 medium. When inoculated, the residual glycerol could potentially be used by *M. sedula*. Glycerol degradation has been described in halophilic Archaea (Williams et al., 2017) and further proposed in *Sulfolobus acidocaldarius* by Schmerling et al. (2024). Additionally, D-glyceraldehyde-3-phosphate is released in the final step of tryptophan biosynthesis (Tang et al., 2000) and serves as the initial precursor of thiamine biosynthesis (Zaparty et al., 2010).

Archaea in the order Sulfolobales are known for using a modified Entner-Doudoroff (ED) pathway for glucose metabolism. Specifically, utilizing D-gluconate/galactonate, 2-keto-3-deoxygluconate and 2-keto-3-deoxy-6-phosphogluconate, as previously reported for *S. solfataricus*, *S. acidocaldarius* and *Metallosphaera* spp. (Lamble et al., 2003; Nunn et al., 2010; Kim and Lee, 2006; Wang et al., 2020). In hyperthermophilic Archaea, non-, branched-, and semi-phosphorylative ED modifications have been identified (De Rosa et al., 1984; Budgen and Danson, 1986; Selig et al., 1997; Siebers and Schönheit, 2005; Reher and Schönheit, 2006; Sutter et al., 2016). While 2-keto-3-deoxygluconate is used as an intermediate in all three ED pathways, 2-keto-3-deoxy-6-phosphogluconate is involved only in branched and semi-phosphorylative ED (Siebers and Schönheit, 2005; Sutter et al., 2016). However, *S. solfataricus* from the order Sulfolobales utilizes a branched ED pathway (Ahmed et al., 2005). This is congruent with our findings for *M. sedula*, which shares the same order (Sulfolobales) and highlights the usage of unusual sugar degradation pathways in Archaea.

Among sulfur metabolism-related compounds, decylubiquinol is produced from decylubiquinone by enzymes expressed from the sulfur reduction gene cluster (DoxD) (Kletzin et al., 2004; Müller et al., 2004; Auernik and Kelly, 2008), whereas MoCo II is associated with sulfite:acceptor oxidoreductase (SAOR) in *Metallosphaera* spp. (Liu et al., 2014, 2021).

In terms of microbial interactions, cell surface interactions require direct contact resulting in biofilm formation (Lewis et al., 2023). This is mediated by carbohydrates, including hexose, N-acetyl-D-glucosamine/galactosamine, and N-acetylmuramic acid (Koerdt et al., 2010, 2012). Cell–cell interactions, such as quorum sensing, do not require direct cell contact, but relay on messenger molecules (Charlesworth et al., 2020; Prescott and Decho, 2020). We detected N-(3-oxohexanoyl)-L-homoserine lactone, and its indicative lactone ring, as evidence of acyl-homoserine lactone (AHL) quorum sensing, which were characterized in *S. solfataricus* and *S. islandicus* (Ng et al., 2011; Hiblot et al., 2012). Few examples of quorum sensing have been reported only in halophilic and methanogenic Archaea so far (Tommonaro et al., 2012; Zhang et al., 2012).

Of all the identified metabolites, a subgroup can be seen to support the adaptation of *M. sedula* to extreme environments. Two of the detected amino acids, histidine and methionine, have been shown to be involved in tolerance and resistance to copper toxicity in *M. sedula* (Auernik and Kelly, 2008). Leucylvaline can support the growth of *S. islandicus*, promoting its adaptation to extreme and fluctuating environmental conditions in volcanic hot spring habitats (Weitzel et al., 2020). Furthermore, S-adenosylmethionine, a cofactor of methyl transferases, was detected in *M. sedula* in this study. This cofactor was also found in *S. solfataricus* (Cacciapuoti et al., 1996) and *S. acidocaldarius* (Zeng et al., 2018). In extremophiles, S-adenosylmethionine may assist in protein methylation, leading to a higher resistance to aggregation and denaturation at physiological pH compared to the unmethylated form, and increasing the stability of proteins in high-temperature environments, as shown for *S. solfataricus* (Febbraio et al., 2004).

8-amino-7-oxononanoic acid was downregulated in acid stress tolerance experiments with the bioleaching microorganism *Acidithiobacillus caldus*. It was proposed that *A. caldus* utilizes acid resistance mechanisms via the formation of extracellular polymeric substances and biofilm formation (Feng et al., 2021). The detected N-glycan building blocks, N-acetyl-D-glucosamine/galactosamine and N-acetylmuramic acid, play different roles in Sulfolobales, as they interact with the environment while maintaining cell shape and supporting cell protection under extreme environmental conditions (Jarrell et al., 2014; Palmieri et al., 2013; Van Wolferen et al., 2020).

Different members of the Sulfolobales order have different compositions in saturated quinones in response to their redox environment. Consequently, the quinone distribution of a given member of the Sulfolobales order can be used to reconstruct environmental redox conditions (Brassell et al., 1986; Hiraishi, 1999; Elling et al., 2016; Becker et al., 2018). Therefore, we focused on the composition of the respiratory chain electron carriers in *M. sedula* and the degree of saturation of quinones as an indicative microbial fingerprint.

#### 3.2.3 Focus on thiophene-bearing quinones

The respiratory chain electron carriers were investigated in more detail in terms of their oxidation states (Table 4). To this end, a mass list containing all possible oxidation states of caldariellaquinones, sulfolobusquinones, and benzodithiophenequinones and their corresponding *m/z* values was created, and molecular assignment was performed using the advanced data analysis software MetaboScape®. The main features for molecular assignment were as follows: assign the corresponding *m/z* values within the dataset (over an intensity threshold of 10,000 counts, minimum peak length of six spectra, and distinct isotopic pattern fit) to the molecules defined in the quinone mass list only when they are present in two separate samples.

**Table 4:** Analytical parameters used for identification of caldariellaquinones (CQ), sulfolobusquinones (SQ), and benzodithiophenequinones (BDTQ) were identified using Metaboscape®.

RT [min]	Observed *m/z*	Theoretical *m/z*	Ions	Observed M	*Δm/z* [mDa]	*Δm/z* [ppm]	mSigma	Average integrated peak area	[%]	Molecular formula	Identified compounds
8.91	581.34476	581.34574	[M + Na]^+^	558.35554	−0.98	−1.685	45.4	36,712	6.9	C_34_H_54_O_2_S_2_	CQ_5:1_
10.84	511.26727	511.26749	[M + Na]^+^	488.27805	−0.22	−0.433	55.2	13,773	2.6	C_29_H_44_O_2_S_2_	CQ_4:1_
14.07	459.32844	459.32913	[M + H]^+^	458.32116	−0.69	−1.505	33.3	14,299	2.7	C_29_H_46_O_2_S	SQ_4:0_
15.10	457.31365	457.31348	[M + H]^+^	456.30637	0.17	0.377	34.5	357,336	66.7	C_29_H_44_O_2_S	SQ_4:1_
15.41	529.40689	529.40738	[M + H]^+^	528.39961	−0.49	−0.927	43.8	47,033	8.8	C_34_H_56_O_2_S	SQ_5:0_
15.96	527.39113	527.39173	[M + H]^+^	526.38385	−0.60	−1.133	53.4	29,716	5.5	C_34_H_54_O_2_S	SQ_5:1_
15.93	543.33195	543.33250	[M + H]^+^	542.32467	−0.55	−1.014	58.4	36,571	6.8	C_33_H_50_O_2_S_2_	BDTQ_5:0_

The mSigma corresponds to the isotopic pattern fit of the software.

Thiophene-bearing quinones of *M. sedula* (Table 4) were analyzed and identified in the form of oxidized caldariellaquinones (CQ_4:1_ and CQ_5:1_), sulfolobusquinones (SQ_4:0_, SQ_4:1_, SQ_5:0_, and SQ_5:1_), and benzodithiophenequinones (BDTQ_5:0_). Caldariellaquinones were first described by De Rosa et al. (1977) and adapted by organisms thriving in extreme environments (pH 1.4–2.6; 75–89°C) with a corresponding durable membrane structure found in *Sulfolobus* and *Acidianus* spp. (De Rosa and Gambacorta, 1988). Subsequently, caldariellaquinones have been reported in *S. solfataricus*, and later in *M. sedula* (De Rosa et al., 1983a, 1983b; Lanzotti et al., 1986; Huber et al., 1989), while benzodithiophenequinones have been identified in *S. solfataricus* (Collins and Langworthy, 1983; Trincone et al., 1986, 1992; Lanzotti et al., 1986). Variations among the produced CQ, SQ, and BDTG molecules are correlated with the presence of oxygen during growth (Trincone et al., 1989; Nicolaus et al., 1992). For the order of Sulfolobales, Elling et al. (2016) showed a distribution of CQ_6:0_ (86.1%), CQ_6:1_ (12.2%) for *S. acidocaldarius*, CQ_6:0_ (85.8%), CQ_6:1_ (13.7%) for *S. solfataricus*, and SQ_6:0_ (42.9%), CQ_6:0_ (36.4%), and CQ_6:1_ (14.6%) for *S. islandicus* as major quinone components, with traces of BDTQ_6:0_ (0.4%) only found in *S. islandicus.* An average semiquantitative distribution among the biological triplicates based on integrated peak area showed percentages of SQ_4:0_ (2.7), SQ_4:1_ (66.7), SQ_5:0_ (8.8), SQ_5:1_ (5.5), CQ_4:1_ (2.6), CQ_5:1_ (6.9), and BDTQ_5:0_ (6.8). Our findings present a shift from CQ to SQ, with SQ_4:1_ being the most abundant, as the preferred quinone with traces of BDTQ. For all three quinones, the detection limit was <2 ppm. These quinones differ in the primary ions detected, i.e., H^+^ for SQ and BDTQ, and Na^+^ for CQ. Comparing the identified CQs with the SQs, the retention time was reversed: SQ_5:1_ > SQ_4:1_ but CQ_5:1_ < CQ_4:1_. However, the relative proportions of quinones differ between Sulfolobales species. Therefore, adaptations to environmental conditions may be reflected in SQs, CQs, and BDTQs distributions (Elling et al., 2016). Profiling quinones may enable monitoring of shifts in microbial communities from oxic to anoxic conditions and allow archaeal diversity to be characterized, complementing membrane lipid- and gene-based approaches (Brassell et al., 1986; Hiraishi, 1999; Elling et al., 2016; Becker et al., 2018). Furthermore, thiophene-bearing quinones have been proposed to have potential as biomarker for astrobiological life detection owing to their durability and stability over geological timescales (Eigenbrode et al., 2018; Heinz and Schulze-Makuch, 2020; Geisberger et al., 2021). The mass spectrometry-based analysis conducted in our study confirm that it is possible to detect them in *M. sedula* grown on mineral materials.

### 3.3 Perspectives on environmental metabolomics

In this study, we provided a protocol for the analysis of metabolites from one species of Archaea grown on mineral materials in a laboratory setting. Metabolomics-based technologies have been shown to be useful to monitor biological responses in environmental studies (for examples, see Sardans et al., 2011; Chandran et al., 2020). To set the stage for environmental field studies of chemolithotrophic extremophiles where multiple species and minerals are present, one could perform experiments as an intermediate step, such as monitoring different species, or varying mineral substrates.

Moreover, acidophilic, chemolithotrophic, iron- and sulfur-oxidizing microorganisms are major players in biomining processes and can be used to recover various metals from copper-, uranium-, and gold-bearing minerals and mineral concentrates (Rawlings, 2002, 2005). As the metal recovery rate of biomining is directly linked to the microbial communities involved, multi-omics approaches could be applied to explore microbial diversity, metabolic characteristics, and resistance mechanisms in extreme environments. This way, the corresponding genes, enzymes, metabolites, and active metabolic pathways can be identified (Li and Wen, 2021). With our protocol, it was possible to identify metabolites and draw hypotheses about the metabolic pathways they are involved in, which could at some point lead to similar characterization for microorganisms with the potential for biomining operations.

## 4 Conclusion

To overcome the challenges associated with the extraction of microbial organic molecules from mineral materials, an improved extraction protocol has been devised and applied. By implementing a metabolomic approach, an overview of the metabolome of the iron- and sulfur-oxidizing archaeon *M. sedula* was proposed. We successfully identified key metabolites of *M. sedula* and ascribed them to their metabolic pathways. We detected molecules indicative of cell surface interactions involved in biofilm formation and acyl-homoserine lactone (AHL) quorum sensing signaling molecules involved in cell–cell communication. Moreover, we successfully analyzed and identified different saturated thiophene-bearing quinones in *M. sedula*. These metabolites are stable, resistant, and preservable biomarker under extreme conditions and can be preserved and extracted in many extreme environmental scenarios. The efficiency of this protocol may be limited by the differences in the solubility of the target molecules. This could be circumvented by adapting the solvents chosen for liquid–liquid extraction. The present study further demonstrates the possibility of extracting metabolites from metallophilic Archaea, paving the way for metabolomics or even multi-omics investigations of microbe-mineral interactions in a number of biotechnological, environmental, and astrobiological applications.

## Data Availability

The original contributions presented in the study are included in the article/Supplementary material, further inquiries can be directed to the corresponding author/s.
