# Supplementary material for: Improved protocol for metabolite extraction and identification of respiratory quinones in extremophilic Archaea grown on mineral materials
Source: Front Microbiol. 2025 Jan 8;15:1473270. doi: 10.3389/fmicb.2024.1473270 (PMC11750793; doi:10.3389/fmicb.2024.1473270)
Supplement: Supplementary file 1 [file Data_Sheet_1.docx]

**Supplementary material for “Improved protocol for metabolite extraction and identification of respiratory quinones in extremophilic Archaea grown on mineral materials”**

Sebastian V Gfellner^1,2^, Cyril Colas^1,2,3^, Guillaume Gabant^1,2^, Janina Groninga^4^, Martine Cadene^1,2^, and Tetyana Milojevic^1,2*^

^1^UPR4301 Centre de biophysique moléculaire (CBM), Orléans, France

^2^Université d'Orléans, Orléans, France

^3^UMR7311 Institut de Chimie Organique et Analytique (ICOA), Orléans, France

^4^Center for Marine Environmental Sciences, University of Bremen, Bremen, Germany

*** Correspondence:**Tetyana Milojevic
tetyana.milojevic@cnrs-orleans.fr

**List of supplementary figure and tables:**

**- Supplementary Figure S1**

**- Supplementary Table S1**

**- Supplementary Table S2**


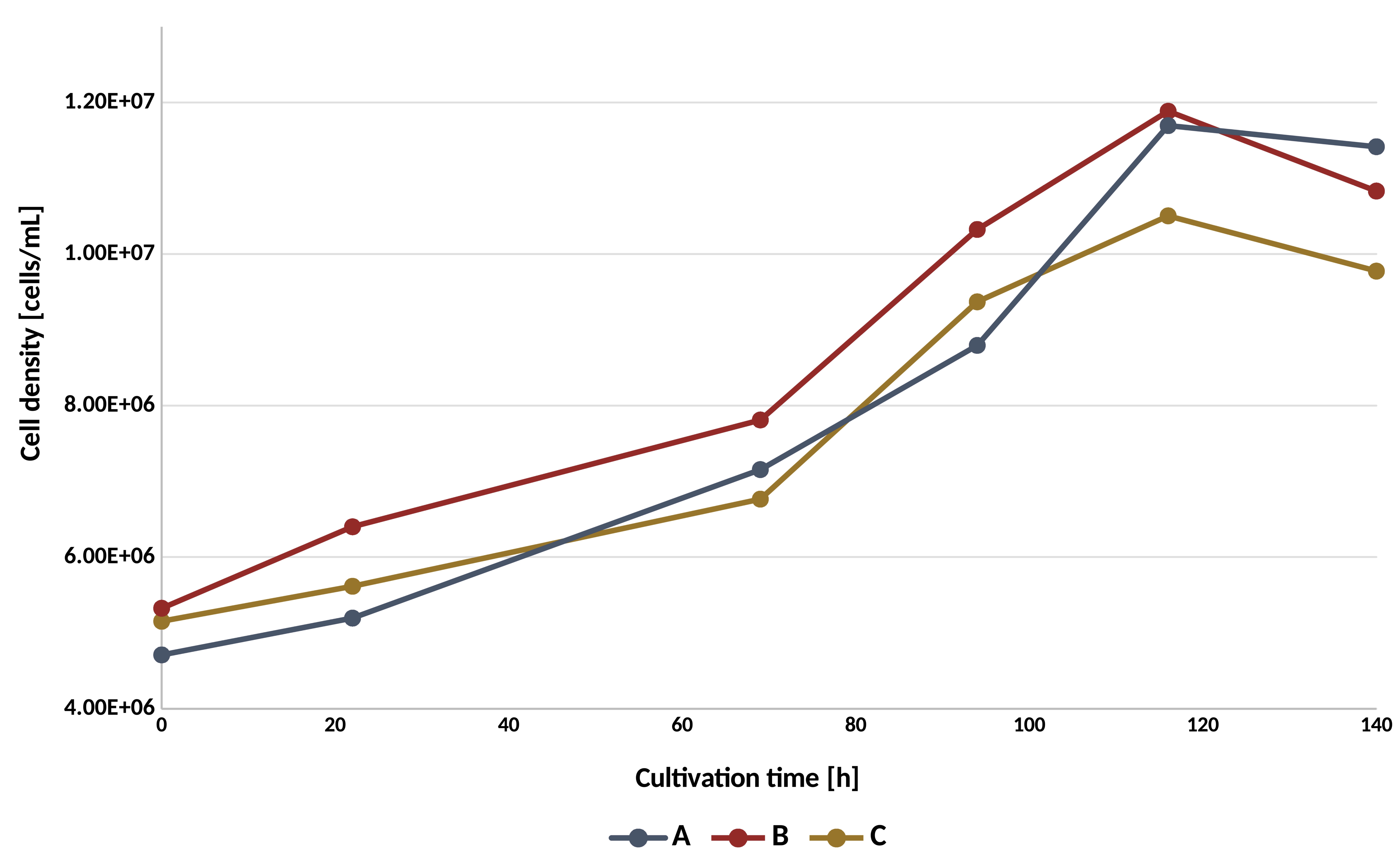


**Supplementary Figure S1.** Cell density [cells/mL] of n = 3 biological replicates (A, B, and C) from inoculation (t = 0 h) to harvest (t = 140 h).
